# Molecular diversity of *Cotton leaf curl Gezira virus *isolates and their satellite DNAs associated with okra leaf curl disease in Burkina Faso

**DOI:** 10.1186/1743-422X-7-48

**Published:** 2010-02-23

**Authors:** Fidèle Tiendrébéogo, Pierre Lefeuvre, Murielle Hoareau, Julie Villemot, Gnissa Konaté, Alfred S Traoré, Nicolas Barro, Valentin S Traoré, Bernard Reynaud, Oumar Traoré, Jean-Michel Lett

**Affiliations:** 1 Laboratoire de Biochimie & Biologie Moléculaire, CRSBAN/UFR/SVT, Université de Ouagadougou 03 BP 7021 Ouagadougou 03, Burkina Faso; 2 CIRAD, UMR 53 PVBMT CIRAD-Université de la Réunion, Pôle de Protection des Plantes, 7 Chemin de l'IRAT, 97410 Saint Pierre, La Réunion, France; 3 Institut de l'Environnement et de Recherches Agricoles (INERA) 01 BP 476 Ouagadougou 01, Burkina Faso

## Abstract

Okra leaf curl disease (OLCD) is a major constraint on okra (*Abelmoschus esculentus*) production and is widespread in Africa. Using a large number of samples representative of the major growing regions in Burkina Faso (BF), we show that the disease is associated with a monopartite begomovirus and satellite DNA complexes. Twenty-three complete genomic sequences of *Cotton leaf curl Gezira virus *(CLCuGV) isolates associated with OLCD, sharing 95 to 99% nucleotide identity, were cloned and sequenced. Six betasatellite and four alphasatellite (DNA-1) molecules were also characterized. The six isolates of betasatellite associated with CLCuGV isolates correspond to *Cotton leaf curl Gezira betasatellite *(CLCuGB) (88 to 98% nucleotide identity). One isolate of alphasatellite is a variant of Cotton leaf curl Gezira alphasatellite (CLCuGA) (89% nucleotide identity), whereas the three others isolates appear to correspond to a new species of alphasatellite (CLCuGA most similar sequence present 52 to 60% nucleotide identity), provisionally named Okra leaf curl Burkina Faso alphasatellite (OLCBFA). Recombination analysis of the viruses demonstrated the interspecies recombinant origin of all CLCuGV isolates, with parents being close to *Hollyhock leaf crumple virus *(AY036009) and *Tomato leaf curl Diana virus *(AM701765). Combined with the presence of satellites DNA, these results highlight the complexity of begomoviruses associated with OLCD.

## Findings

Okra leaf curl disease (OLCD) is commonly observed among okra (*Abelmoschus esculentus*) crops in Burkina Faso (BF) and several African countries [1-5]. Affected plants are severely stunted with apical leaf curl (upward or downward), distortion and thickening of the veins. In BF, okra is widely grown in both rainy and dry seasons. It is a major source of income particularly for small-scale farming. Viral diseases are important constraints in the production of this crop [6]. Recently, it was shown that OLCD in Africa is associated with a complex of begomoviruses: *Cotton leaf curl Gezira virus *(CLCuGV; [7,4,5]), *Okra yellow crinkle virus *(OYCrV; [8]) and *Hollyhock leaf crumple virus *(HoLCrV;[9,10]).

Viruses of the genus *Begomovirus *belong to the family *Geminiviridae *and are transmitted by the whitefly vector *Bemisia tabaci *to dicotyledonous plants [11]. They have emerged as a major constraint for many vegetable and fibre crops throughout the world [12]. Begomoviruses are either bipartite with two genomic components, designated as DNA-A and DNA-B or monopartite with only DNA-A like components [13]. Some of the monopartite begomoviruses are also associated with additional circular ssDNA molecules, such as betasatellite or alphasatellite (previously known as DNA-1) that are nearly half the size of DNA-A. Betasatellites have been involved in pathogenicity but alphasatellites have no known function and are certainly not involved in symptom induction [14-16]. Alphasatellites have only been shown to be present in plants infected with monopartite begomoviruses in association with betasatellites [17].

The aim of our study was to characterize at the molecular level the complex of viruses involved in OLCD in BF and their relationship with other begomoviruses. In association with a single Old World begomovirus, we describe their associated satellite DNAs.

During May 2008 to April 2009, 74 leaf samples exhibiting typical OLCD symptoms were collected from okra fields in the major growing regions of BF around Tiébélé, Kampala, Pô, Kamboinsé, Bazèga and Bama (Kou valley) localities. Total DNA was extracted using DNeasy^® ^Plant Minikit (Qiagen) before detection of begomoviruses using polymerase chain reaction (PCR) with specific primers of either the DNA-A [18] or betasatellite and alphasatellite [19,20]. Full-length viral genomes were amplified from the PCR-positive samples by rolling-circle amplification (RCA) [21]. The amplified DNAs were digested with endonucleases *Bam*HI or *Pst*I, and the DNA fragments of the expected size (~2.8 kb for DNA-A and ~1.4 kb for satellites) were cloned into pGEM^®^-3Zf (+) vector (Promega Biotech). Cloned genome components were sequenced by Macrogen Inc. (South Korea). Contigs were assembled with the DNAMAN software (Lynnon, Quebec, Canada) and subsequently aligned using the ClustalW tool [22] implemented in MEGA 4 [23]. Sequence comparisons were performed in MEGA 4 with pairwise deletion of gaps. The optimal model of sequence evolution, defined with ModelTest [24], was used for maximum likelihood (ML) phylogenetic reconstruction using PHYML_v2.4.4 [25]. The degree of support for individual branches within the resulting phylogenetic trees was assessed with 1000 full ML bootstrap iterations. The trees were visualized using FigTree v1.1.1 software.

Recombination was analyzed using our sequences and a set of sequences representing the whole African begomovirus diversity (representing an alignment of 121 sequences). Detection of potential recombinant sequences, identification of likely parental sequences, and localization of possible recombination breakpoints was carried out using RDP [26], GENECONV [27], BOOTSCAN [28], MAXIMUM CHI SQUARE [29], CHIMAERA [28], SISTER SCAN [30] and 3Seq [31] recombination detection methods as implemented in RDP3 [32]. The analysis was performed with default settings for the different detection methods and a Bonferroni corrected *P*-value cut-off of 0.05. Only events detected with 3 methods or more were accepted.

Despite a very poor preservation of samples (high necrosis), 48 samples of the 74 were detected as being infected with begomovirus using PCR amplifications with the universal primer pair VD360-CD1266 recovering the conserved CP ORF [18]. From the positive samples, 23 begomovirus genome sequences with length between 2761 to 2773 nucleotides (nt) were successfully obtained using RCA. Pairwise sequence comparison demonstrated that the 23 new genome sequences of monopartite begomoviruses from BF are genetically related to the same strain (94.7 to 100% identity amongst themselves). A BLAST search identify the most similar virus sequences as being *Cotton leaf curl Gezira virus *(CLCuGV) isolates, a monopartite *Begomovirus *first identified in Sudan [33]. Further pairwise sequence analyses showed that the 23 sequences shared between 94.8 to 98.8% nt identity with CLCuGV isolates from Niger (FJ469626, EU432373, EU432374), 93.7 to 96.2% with CLCuGV from Sudan (AY036007, AY036008), 92.4 to 96.1% with CLCuGV from Egypt (AY036006, AY036010) and 89.3 to 91.4% with CLCuGV from Cameroon (FM164726). According to the ICTV guidelines, these results of nucleotide identity <93% between isolates of CLCuGV suggest the existence of several strains within this begomovirus species. Similar comparisons performed with the other two *Begomovirus *species infecting *Malvaceae *in Africa showed low nucleotide sequence identity: 71.7 to 72.6% obtained with *Okra yellow crinkle virus *(DQ902715, EU024118, FM164724) and 83.4 to 84.2 with *Hollyhock leaf crumple virus *(AY036009, AF014881).

All 23 isolates of begomovirus infecting okra in BF have the typical genome organization of Old World monopartite begomoviruses. This organization consisted of the presence of six open reading frames (ORFs) on the DNA-A corresponding to V1 and V2 on the virion strand and C1, C2, C3 and C4 on the complementary strand [34]. The IR sequences located between the start codons of the C1 and V2 are 289 to 300 nt. In this region, they present a typical replication origin (↓), including an inverted repeat sequence containing the highly conserved nanonuclotide sequence TAATATT↓AC [35,5].

**Figure 1:**
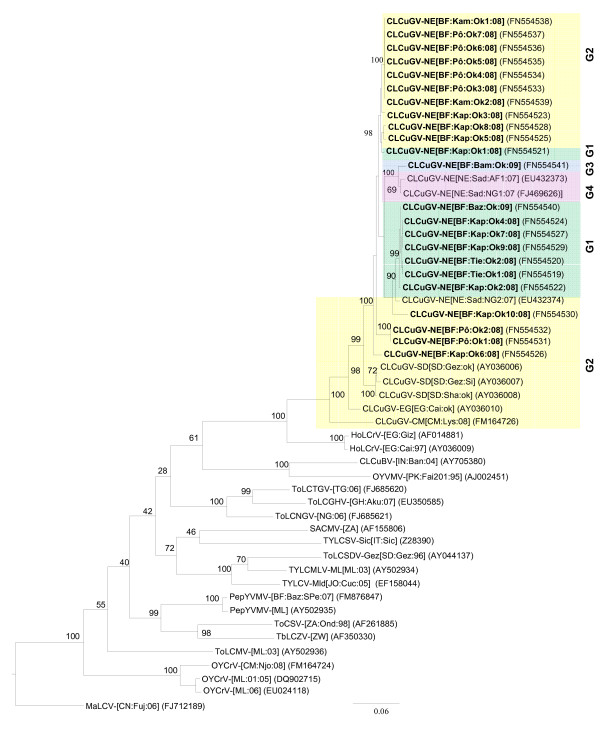
**Maximum likelihood tree based on the complete DNA-A sequences of twenty-three *Cotton leaf curl Gezira virus *isolates from Burkina Faso (in bold; see **Table 2 **for isolates name and acronyms), plus additional sequences from African and Asian monopartite and bipartite begomoviruses**. *Begomovirus *acronyms used are *Cotton leaf curl Gezira virus *(CLCuGV), *Hollyhock leaf crumple virus *(HoLCrV), *Cotton leaf curl Bangalore virus *(CLCuBV), *Okra yellow vein mosaic virus *(OYVMV), *Tomato leaf curl Togo virus *(ToLCTGV), *Tomato leaf curl Ghana virus *(ToLCGHV), *Tomato leaf curl Nigeria virus *(ToLCNGV), *South African cassava mosaic virus *(SACMV), *Tomato yellow leaf curl Sardinia virus *(TYLCSV), *Tomato leaf curl Sudan virus *(ToLCSDV), *Tomato yellow leaf curl Mali virus *(TYLCMLV), *Tomato yellow leaf curl virus*-Mild (TYLCV-Mld), *Pepper yellow vein Mali virus *(PepYVMV), *Tomato curly stunt virus *(ToCSV), *Tobacco leaf curl Zimbabwe virus *(TbLCZV), *Tomato leaf curl Mali virus *(ToLCMV), *Okra yellow crinkle virus *(OYCrV) and *Malvastrum leaf curl virus *(MaLCV). For the complete description of isolate descriptors refer to Fauquet et al. (2008). Four genetic groups (G1 to G4) have been defined on the presence or absence of recombination events (Figure 4), and are represented here.

Based on the presently applicable species demarcation threshold of 89% for begomoviruses [36], we conclude that the 23 begomovirus isolates isolated from okra in BF belong to the species *Cotton leaf curl Gezira virus *and the Niger strain (See Table 1 for percentage of similarities and Table 2 for isolates description and accession numbers). In addition, a maximum-likelihood phylogenetic tree constructed using PHYML and the GTR+I+G model of sequence evolution (ModelTest), confirms that okra begomoviruses reported here cluster with the isolates of *Cotton leaf curl Gezira virus *(CLCuGV) (Figure 1). A clear phylogeographic separation is observed between the diversity of CLCuGV isolates of okra: West Africa (Niger strain), Central Africa (Cameroon strain), East Africa (Sudan strain) and north-east of the Africa (Egypt strain).

**Table 1:** Matrix of pairwise identity percentages between the complete DNA-A nucleotide sequences of the twenty-three isolates of okra-infecting *Cotton leaf curl Gezira virus *(CLCuGV) isolates from Burkina Faso and representatives of some begomoviruses infecting okra in Africa.

		CLCuGV-NE [NE:Sad: NG1:07]*	CLCuGV- NE [NE:Sad: AF1:07]*	CLCuGV-NE [NE:Sad: NG2:07]*	CLCuGV-EG [EG:Cai: Ok]*	CLCuGV-SD [SD:Sha: Ok]*	CLCuGV- SD [SD:Gez: Si]	CLCuGV-SD [SD:Gez: Ok]*	CLCuGV-CM [CM:Lys: Ok:08]*	OYCrV-[ML:06]*	OYCrV-[CM:Njo: Ok:08]*	OYCrV-[ML:01: 05]*	HoLCrV-[EG:Cai:97]	HoLCrV-[EG:Giz]
NE [BF:Tie:Ok1:08]*		**96.3**	**96.8**	**98.7**	**93.3**	**95**	**94.6**	**94.8**	**89.6**	72.4	72.6	72.4	84.1	83.9
NE [BF:Tie:Ok2:08]*		**96.3**	**96.8**	**98.7**	**93.3**	**95**	**94.6**	**94.8**	**89.6**	72.4	72.4	72.4	84.1	83.9
NE [BF:Kap:Ok1:08]*		**97.3**	**96.8**	**96.8**	**94.4**	**96.1**	**95.9**	**96.0**	**91.4**	72.0	72.4	72.0	83.7	83.7
NE [BF:Kap:Ok2:08]*		**96.4**	**96.4**	**98.8**	**93.3**	**94.9**	**94.6**	**94.8**	**89.3**	72.2	72.3	72.2	83.9	83.7
NE [BF:Kap:Ok3:08]*		**96.8**	**97.1**	**96.7**	**94.4**	**96.2**	**95.9**	**96.0**	**91.4**	72.1	72.6	72.0	84.1	84.1
NE [BF:Kap:Ok4:08]*		**96.3**	**96.8**	**98.7**	**93.3**	**95.0**	**94.7**	**94.8**	**89.6**	72.4	72.6	72.4	84	83.8
NE [BF:Kap:Ok5:08]*		**96.6**	**96.2**	**96.8**	**94.2**	**96.1**	**95.9**	**96.1**	**91.2**	72	72.3	71.9	83.5	83.5
NE [BF:Kap:Ok6:08]*		**95.9**	**96.2**	**96.2**	**94.6**	**96.2**	**95.8**	**96.1**	**91.5**	71.7	72.4	71.7	84.0	84.1
NE [BF:Kap:Ok7:08]*		**96.3**	**96.8**	**98.6**	**93.2**	**95**	**94.6**	**94.8**	**89.6**	72.3	72.5	72.3	83.9	83.7
NE [BF:Kap:Ok8:08]*		**96.8**	**96.4**	**96.7**	**94.3**	**96.1**	**95.9**	**96.0**	**91.3**	72.0	72.4	71.9	83.8	83.7
NE [BF:Kap:Ok9:08]*		**96.3**	**96.8**	**98.7**	**93.3**	**95.0**	**94.6**	**94.8**	**89.6**	72.4	72.6	72.3	83.9	83.8
NE [BF:Kap:Ok10:08]*		**95.0**	**94.8**	**97.3**	**93.2**	**94.8**	**94.6**	**94.7**	**89.3**	72.2	72.0	72.2	83.6	83.4
NE [BF:Pô:Ok1:08]*		**95.4**	**95.1**	**95.9**	**94.0**	**95.7**	**95.4**	**95.6**	**90.7**	71.9	72.3	71.9	83.4	83.4
NE [BF:Pô:Ok2:08]*		**95.3**	**95.1**	**95.8**	**94.0**	**95.7**	**95.4**	**95.6**	**90.6**	71.9	72.3	71.9	83.4	83.4
NE [BF:Pô:Ok3:08]*		**96.6**	**96.5**	**96.5**	**94.4**	**95.9**	**95.7**	**95.8**	**91.1**	72	72.3	71.9	83.8	83.8
NE [BF:Pô:Ok4:08]*		**96.6**	**96.5**	**96.5**	**94.4**	**95.9**	**95.7**	**95.8**	**91.1**	72	72.3	71.9	83.8	83.8
NE [BF:Pô:Ok5:08]*		**96.6**	**96.5**	**96.5**	**94.4**	**95.9**	**95.7**	**95.8**	**91.1**	72	72.3	71.9	83.8	83.8
NE [BF:Pô:Ok6:08]*		**96.6**	**96.5**	**96.5**	**94.4**	**95.9**	**95.7**	**95.8**	**91.1**	72	72.3	71.9	83.8	83.8
NE [BF:Pô:Ok7:08]*		**96.6**	**96.5**	**96.5**	**94.4**	**95.9**	**95.7**	**95.8**	**91.1**	72	72.3	71.9	83.8	83.8
NE [BF:Kab:Ok1:08]*		**96.6**	**96.5**	**96.5**	**94.4**	**95.9**	**95.7**	**95.8**	**91.1**	72	72.3	71.9	83.8	83.8
NE [BF:Kab:Ok2:08]*		**96.6**	**96.5**	**96.5**	**94.4**	**95.9**	**95.7**	**95.8**	**91.1**	72	72.3	71.9	83.8	83.8
NE [BF:Baz:Ok:09]*		**96.4**	**96.7**	**98.8**	**93.4**	**95.1**	**94.7**	**95**	**89.6**	72.4	72.4	72.4	83.9	83.7
NE [BF:Bam:Ok:09]*		**98.7**	**98.2**	**95.9**	**92.4**	**93.9**	**93.7**	**93.8**	**89.3**	72.4	72.7	72.3	84.2	84.0

*Begomovirus DNA-A isolated on Okra (*Abelmoshus esculentus*) from Burkina Faso

Acronyms: Niger: NE; Burkina Faso: BF; Tiébélé: Tie; Kampala: Kap; Kamboinsé: Kab; Bazéga: Baz; Bama: Bam

Percentages of identity over the 89% taxonomic threshold of begomovirus are presented in bold

**Table 2:** Name, acronym and accession numbers of begomovirus reported in this study.

Virus name	Acronym	Isolates groups	DNA-A length (nt)	Accession numbers
Cotton leaf curl Gezira virus-Niger [Burkina Faso:Tiébélé:Okra1:2008]	CLCuGV-NE [BF:Tie:Ok1:08]	G1	2762	FN554519
Cotton leaf curl Gezira virus-Niger [Burkina Faso:Tiébélé:Okra2:2008]	CLCuGV-NE [BF:Tie:Ok2:08]	G1	2762	FN554520
Cotton leaf curl Gezira virus-Niger [Burkina Faso:Kampala:Okra1:2008]	CLCuGV-NE [BF:Kap:Ok1:08]	G1	2761	FN554521
Cotton leaf curl Gezira virus-Niger [Burkina Faso:Kampala:Okra2:2008]	CLCuGV-NE [BF:Kap:Ok2:08]	G1	2762	FN554522
Cotton leaf curl Gezira virus- Niger [Burkina Faso:Kampala:Okra3:2008]	CLCuGV-NE [BF:Kap:Ok3:08]	G2	2763	FN554523
Cotton leaf curl Gezira virus-Niger [Burkina Faso:Kampala:Okra4:2008]	CLCuGV-NE [BF:Kap:Ok4:08]	G1	2762	FN554524
Cotton leaf curl Gezira virus-Niger [Burkina Faso:Kampala:Okra5:2008]	CLCuGV-NE [BF:Kap:Ok5:08]	G2	2762	FN554525
Cotton leaf curl Gezira virus-Niger [Burkina Faso:Kampala:Okra6:2008]	CLCuGV-NE [BF:Kap:Ok6:08]	G2	2763	FN554526
Cotton leaf curl Gezira virus-Niger [Burkina Faso:Kampala:Okra7:2008]	CLCuGV-NE [BF:Kap:Ok7:08]	G1	2762	FN554527
Cotton leaf curl Gezira virus-Niger [Burkina Faso:Kampala:Okra8:2008]	CLCuGV-NE [BF:Kap:Ok8:08]	G2	2762	FN554528
Cotton leaf curl Gezira virus-Niger [Burkina Faso:Kampala:Okra9:2008]	CLCuGV-NE [BF:Kap:Ok9:08]	G1	2765	FN554529
Cotton leaf curl Gezira virus-Niger [Burkina Faso:Kampala:Okra10:2008]	CLCuGV-NE [BF:Kap:Ok10:08]	G2	2773	FN554530
Cotton leaf curl Gezira virus-Niger [Burkina Faso:Pô:Okra1:2008]	CLCuGV-NE [BF:Pô:Ok1:08]	G2	2770	FN554531
Cotton leaf curl Gezira virus-Niger [Burkina Faso:Pô:Okra2:2008]	CLCuGV-NE [BF:Pô:Ok2:08]	G2	2770	FN554532
Cotton leaf curl Gezira virus-Niger [Burkina Faso:Pô:Okra3:2008]	CLCuGV-NE [BF:Pô:Ok3:08]	G2	2761	FN554533
Cotton leaf curl Gezira virus-Niger [Burkina Faso:Pô:Okra4:2008]	CLCuGV-NE [BF:Pô:Ok4:08]	G2	2761	FN554534
Cotton leaf curl Gezira virus-Niger [Burkina Faso:Pô:Okra5:2008]	CLCuGV-NE [BF:Pô:Ok5:08]	G2	2761	FN554535
Cotton leaf curl Gezira virus-Niger [Burkina Faso:Pô:Okra6:2008]	CLCuGV-NE [BF:Pô:Ok6:08]	G2	2761	FN554536
Cotton leaf curl Gezira virus-Niger [Burkina Faso:Pô:Okra7:2008]	CLCuGV-NE [BF:Pô:Ok7:08]	G2	2761	FN554537
Cotton leaf curl Gezira virus-Niger [Burkina Faso:Kamboinsé:Okra1:2008]	CLCuGV-NE [BF:Kab:Ok1:08]	G2	2761	FN554538
Cotton leaf curl Gezira virus-Niger [Burkina Faso:Kamboinsé:Okra2:2008]	CLCuGV-NE [BF:Kab:Ok2:08]	G2	2761	FN554539
Cotton leaf curl Gezira virus-Niger [Burkina Faso:Bazega:Okra:2009]	CLCuGV-NE [BF:Baz:Ok:09]	G1	2762	FN554540
Cotton leaf curl Gezira virus-Niger [Burkina Faso:Bama:Okra:2009]	CLCuGV-NE [BF:Bam:Ok:09]	G3	2771	FN554541

*GenBank-EMBL-DDBJ data bases

**Figure 2:**
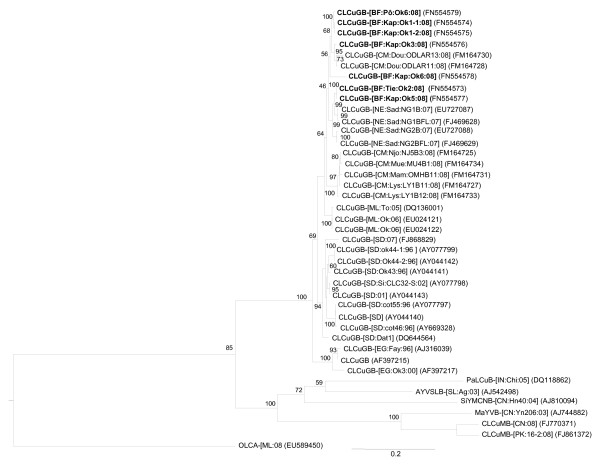
**Maximum likelihood tree based upon alignments of selected sequences of betasatellite genomes**. The betasatellite acronyms used are as described by Briddon et al. [40]: *Cotton leaf curl Gezira betasatellite *(CLCuGB), *Papaya leaf curl betasatellite *(PaLCuB), *Ageratum yellow vein Sri Lanka betasatellite *(AYVSLB), *Sida yellow mosaic China betasatellite *(SiYMCNB), *Malvastrum yellow vein betasatellite *(MaYVB), *Cotton leaf curl Multan betasatellite *(CLCuMB) and Cotton leaf curl alphasatellite (CLCuA-[PK:1:99]) (Outgroup).

Betasatellites were found associated to all isolates from BF except CLCuGV-NE[BF:Baz:Ok:09] and CLCuGV-NE[BF:Bam:Ok:09] while alphasatellites were detected only in association with the seven following isolates: CLCuGV-NE[BF:Kap:Ok7:08], CLCuGV-NE[BF:Pô:Ok1:08], CLCuGV-NE[BF:Pô:Ok2:08], CLCuGV-NE[BF:Pô:Ok4:08], CLCuGV-NE[BF:Pô:Ok5:08], CLCuGV-NE[BF:Pô:Ok6:08] and CLCuGV-NE[BF:Pô:Ok7:08]. Betasatellites associated with CLCuGV-NE[BF:Tie:Ok2:08], CLCuGV-NE[BF:Kap:Ok1:08], CLCuGV-NE[BF:Kap:Ok3:08], CLCuGV-NE[BF:Kap:Ok5:08], CLCuGV-NE[BF:Kap:Ok6:08] and CLCuGV-NE[BF:Pô:Ok6:08] consisted of 1348, 1347, 1349, 1348, 1347 and 1347 nucleotides, respectively. All betasatellites showed typical features consisting of the presence of a single ORF βC1 in the complementary-sense, a region of sequence rich in adenine (A) (nt 703-892 with 58.4 to 58.7% A residues) and a satellite conserved region (SCR) with a predicted stem-loop structure containing the geminivirus nonanucleotide sequence (TAATATTAC) [37]. The nucleotide sequence comparison showed that our sequences had nucleotide identities ranging from 88.1 to 98.7% with betasatellites from Cameroon, Egypt, Mali, Niger and Sudan. In a phylogenetic analysis based upon alignments of the complete betasatellites sequences, the BF betasatellite sequences segregated with betasatellites associated with okra begomoviruses from Africa (Figure 2). Based on the recently established species demarcation threshold for betasatellites (78% nucleotide sequence identity; [38]), the betasatellites reported in this study belong to the same species *Cotton leaf curl Gezira betasatellite *(see Table 3 for betasatellites isolates description and accession numbers). Interestingly and under our knowledge, this species represent the only known betasatellite described in Africa on malvaceous and tomato plants. Associated to the absence of betasatellites in the New World and the existence of a high diversity of betasatellites in Asia, this result confirms that the centre of diversity appears to be in southern Asia [39].

**Table 3:** Betasatelittes and alphasatellites characterized in this study.

		DNA molecule and size (nt)		Accession numbers
Betasatellite and alphasatellite names	Acronym			
		Betasatellite	Alphasatellite	
*Cotton leaf curl Gezira betasatellite*-[Burkina Faso:Tiébélé:Okra2:2008]	CLCuGB- [BF:Tie:Ok2:08]	1348		FN554573
*Cotton leaf curl Gezira betasatellite*-[Burkina Faso:Kampala:Okra1-1:2008]	CLCuGB- [BF:Kap:Ok1-1:08]	1347		FN554574
*Cotton leaf curl Gezira betasatellite*-[Burkina Faso:Kampala:Okra1-2:2008]	CLCuGB- [BF:Kap:Ok1-2:08]	1347		FN554575
*Cotton leaf curl Gezira betasatellite*-[Burkina Faso:Kampala:Okra3:2008]	CLCuGB-[BF:Kap:Ok3:08]	1349		FN554576
*Cotton leaf curl Gezira betasatellite*-[Burkina Faso:Kampala:Okra5:2008]	CLCuGB- [BF:Kap:Ok5:08]	1348		FN554577
*Cotton leaf curl Gezira betasatellite*-[Burkina Faso:Kampala:Okra6:2008]	CLCuGB- [BF:Kap:Ok6:08]	1347		FN554578
*Cotton leaf curl Gezira betasatellite*-[Burkina Faso:Pô:Okra6:2008]	CLCuGB- [BF:Pô:Ok6:08]	1347		FN554579

*Cotton leaf curl Gezira alphasatellite*-[Burkina Faso:Kampala:Okra7:2008]	CLCuGA- [BF:Kap:Ok7:08]		1387	FN554580
Okra leaf curl Burkina Faso alphasatellite-[Burkina Faso:Pô:Okra1:2008]	OLCBFA- [BF:Pô:Ok1:08]		1353	FN554581
Okra leaf curl Burkina Faso alphasatellite-[Burkina Faso:Pô:Okra4:2008]	OLCBFA- [BF:Pô:Ok4:08]		1299	FN554582
Okra leaf curl Burkina Faso alphasatellite-[Burkina Faso:Pô:Okra5:2008]	OLCBFA- [BF:Pô:Ok5:08]		1353	FN554583

*GenBank-EMBL-DDBJ data bases

**Figure 3:**
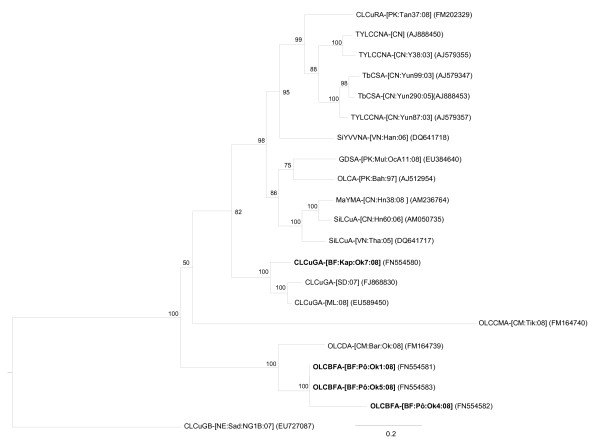
**Maximum likelihood tree based on selected alphasatellite sequences**. Acronyms used are as described by Mubin et al. [17]: Malvastrum yellow mosaic alphasatellite (MaYMA), Sida leaf curl alphasatellite (SiLCuA), Gossypium darwinii symptomless alphasatellite (GDSA), Okra leaf curl alphasatellite (OLCA), Cotton leaf curl Rajastan alphasatellite (CLCuRA), Tomato yellow leaf curl China alphasatellite (TYLCCNA), Tobacco curly shoot alphasatellite (TbCSA), Sida yellow vein Vietnam alphasatellite (SiYVVNA), Okra leaf curl alphasatellite (OLCA), Okra leaf curl Cameroon alphasatellite (OLCCMA) and *Cotton leaf curl Gezira betasatellite *(CLCuGB) (outgroup).

The complete nucleotide sequences of alphasatellites associated with CLCuGV-NE[BF:Kap:Ok7:08], CLCuGV-NE[BF:Pô:Ok1:08], CLCuGV-NE[BF:Pô:Ok4:08] and CLCuGV-NE[BF:Pô:Ok5:08] were determined to be 1382, 1353, 1299 and 1353 nt respectively. The alphasatellite sequence associated with CLCuGV-NE[BF:Kap:Ok7:08] display the highest level of nucleotide sequence identity (88.9%) with Cotton leaf curl Gezira alphasatellite from Mali (CLCuGA-[Mali:Bamako]; EU589450). The phylogenetic analysis showed that the alphasatellite associated with CLCuGV-NE[BF:Kap:Ok7:08] segregate with CLCuGA-[Mali:Bamako] and OLCA-[Sudan:2007] (Figure 3) and has an arrangement typical of characterized alphasatellites [40], containing a single ORF in the virion sense, an A-rich region with 51% adenine and a hairpin structure with the loop sequence TAGTATTAC. The alphasatellites associated with CLCuGV-NE[BF:Pô:Ok1:08], CLCuGV-NE[BF:Pô:Ok4:08] and CLCuGV-NE[BF:Pô:Ok5:08] shared between 84.8 to 100% nucleotide sequence identity amongst themselves and only 52.4 to 60.1% with the alphasatellites associated with CLCuGV-NE[BF:Kap:Ok7:08] and those characterized in Mali and Sudan (respectively, CLCuGA-[Mali:Bamako] and OLCD1-[Sudan:2007]). Considering the suggested species demarcation threshold of 83% nucleotide sequence identity for alphasatellites [17], these alphasatellites represent isolates of a new species provisionally named Okra leaf curl Burkina Faso alphasatellite, clustering together in the phylogenetic tree (Figure 3; see Table 3 for aphasatellites accession numbers). These particular alphasatellite isolates contain a single ORF in the virion sense and a predicted hairpin structure with the loop sequence CAGTATTAC.

**Figure 4:**
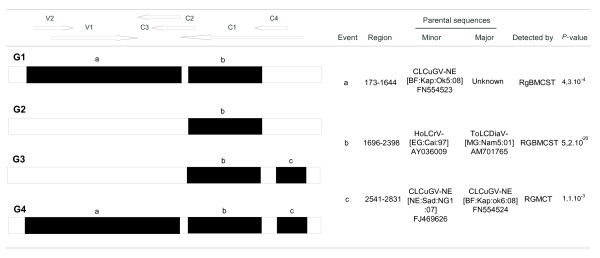
**Recombinant regions (a, b and c) detected within the African isolates of CLCuGV sequences using RDP3**. Four genetic groups (G1 to G4) have been defined on the presence or absence of recombination events. The genome at the top of the figure corresponds to the schematic representation of sequences below. Region coordinates are nucleotide positions of detected recombination breakpoints in the multiple sequence alignment used to detect recombination. Wherever possible, parental sequences are identified. "Major" and "Minor" parents are sequences that were used, along with the indicated recombinant sequence, to identify recombination. Whereas for each identified event the minor parent is apparently the contributor of the sequence within the indicated region, the major parent is the apparent contributor of the rest of the sequence. Note that the identified "parental sequences" are not the actual parents but are simply those sequences most similar to the actual parents in the analysed dataset. Recombinant regions and parental viruses were identified using the RDP (R), GENECONV (G), BOOTSCAN (B), MAXIMUM CHI SQUARE (M), CHIMAERA (C), SISTER SCAN (S) and 3Seq (T) methods. Whereas upper case letters imply a method detected recombination with a multiple comparison corrected P-value < 0.01, lower case letters imply the method detected recombination with a multiple comparison corrected P-value <0.05 but > = 0.01.

Further to the sequence description of the viral isolates, we were interested in their possible recombinant origin. Three distinct recombination events (a, b and c) were detected within the full genome sequences of CLCuGV isolates (Figure 4), using a large sequence alignment of geminiviruses [41]. The presence or absence of these recombination events has identified four genetic groups of viruses (G1 to G4; Figures 1 and 4). Recombination event b present in all CLCuGV isolates involves a major parent being related to the HoLCrV described in north Africa (Egypt; [9]) and a minor parent related to ToLCDiaV described in the south-west Indian Ocean Islands (Madagascar; [41]). Compared to events a and c based on intra-strain recombination, event b seems to be more ancient. The recombination events a and c specific to isolates G1, G3 and G4 have been characterized in Burkina Faso and in Niger and appear to represent a specific geographic signature. The distribution of the recombination breakpoints observed here confirm the existence of recombination hot spots over the intergenic region (IR) and the centre of C1 ORF (Figure 4) as described by Lefeuvre et al. [41]. The recombination event c of isolates G3 and G4 covers the N terminus of the replication associated protein (Rep) which contains the iteron-related domain (IRD) [42]. This domain is involved in the specificity of interaction with iterated DNA motifs (iterons) of the geminivirus origin of replication (*ori*), functioning as essential elements for specific virus replication. Since the IRD domain of G3 and G4 isolates (MAPTKKFRINSKNYFL) is different from the IRD domains of G1 and G2 isolates (MPPSKRFLINAKNYFL or MPFGTHYILSTDILER), the biological aspects of recombination events should be investigated in the future.

In conclusion, in Burkina Faso OLCD is mainly caused by a single begomovirus species and a complex of beta and alpha satellite species, contrary to what happens in the neighbouring countries Mali and Niger (respectively, [5,4]). Taken together, the current molecular results highlight the complex aetiology of the OLCD in Africa and the need for further investigations.

## Competing interests

The authors declare that they have no competing interests.

## Authors' contributions

FT, VSET and OT collected samples; FT, MH, JV cloned and sequenced the viruses and satellites; FT, PL and JML analysed the data and prepared the manuscript. JML, OT, NB, GK, AST, VSET and BR secured funding for the project's execution, and provided ideas and comments during manuscript preparation. All authors have read and approved the final manuscript.
